# Lasalocid inhibits melanoma by down-regulating FOXM1 through PI3K/AKT and JNK/P38 MAPK pathways

**DOI:** 10.7150/jca.101798

**Published:** 2025-01-01

**Authors:** Qiang Yuan, Hangyu Jiang, Maofei Zhu, Yueming Chu, Yan Zhang, Jun Bie, Lin Li

**Affiliations:** 1 Department of Pharmacy, The Second Clinical Medical College of North Sichuan Medical College, Nanchong, Sichuan, China, 637003; 2 School of Pharmacy, North Sichuan Medical College, Nanchong, China, 637100; 3 Department of Pharmacy, The Affiliated Hospital of North Sichuan Medical College, Nanchong, China, 637002; 4 Department of Oncology, The Second Clinical Medical College of North Sichuan Medical College, Nanchong, China, 637003; 5 Institute of Tissue Engineering and Stem Cells, The Second Clinical Medical College of North Sichuan Medical College, Nanchong, China, 637003; 6 Nanchong Key Laboratory of Individualized Drug Therapy, Nanchong, China, 637003

**Keywords:** Lasalocid, FOXM1, PI3K/AKT signaling pathway, JNK/P38 MAPK signaling pathway, Melanoma

## Abstract

Melanoma poses a significant challenge to patients due to its aggressive nature and limited treatment options. Recent studies have suggested that lasalocid, a feed additive ionophore antibiotic, may have potential as an anticancer agent. However, the mechanism of lasalocid in melanoma is unclear. This study found that lasalocid could inhibit melanoma cell proliferation, migration, and invasion, while inducing cell cycle arrest and apoptosis. Transcriptome sequencing and bioinformatics analysis identified FOXM1 as the hub gene of lasalocid-mediated melanoma treatment. *In vitro* experiments confirmed that lasalocid regulates FOXM1 expression through the PI3K/AKT and JNK/P38 MAPK pathways. *In vivo* experiments showed that lasalocid effectively inhibited melanoma growth with acceptable safety. In summary, our study results emphasize lasalocid's potential as a melanoma therapeutic agent and elucidate its role in regulating FOXM1 through the PI3K/AKT and JNK/P38 MAPK pathways.

## Introduction

Melanoma is a highly aggressive form of skin cancer known for its propensity for metastasis, representing a major challenge in oncology. It arises from melanocytes, the pigment-producing cells of the skin, and its malignant behavior poses a considerable barrier to developing effective therapies^1^. According to the Global Cancer Statistics 2018 report, cutaneous melanoma ranks as the 21st most prevalent cancer, comprising 1.6% (287,723) of all newly diagnosed cases and resulting in 0.6% (60,712) of deaths worldwide^2^. Surgical resection, chemotherapy, and radiotherapy continue to serve as the primary modalities in the management of melanoma^3^-^5^. However, the effectiveness of these treatments varies among different cases of melanoma, and the success rates for advanced melanomas are still not ideal, partly due to the emergence of resistance to chemotherapy^6^. The investigation of targeted therapy for melanoma using small molecule drugs is a prominent focus of current research. This approach offers advantages such as reduced side effects compared to conventional treatments like surgery, radiotherapy, and chemotherapy, due to its direct application to tumors. Consequently, the use of small molecule drugs presents an optimal strategy for melanoma treatment. However, there remains a significant number of yet undiscovered and unstudied small molecule drugs in this area, warranting further exploration.

FOXM1 (Forkhead Box M1) is a transcription factor belonging to the FOX family subfamily^7^. FOXM1 is renowned for its critical regulatory functions in various cellular processes, including cell cycle modulation, proliferation, differentiation, apoptosis, and DNA damage repair^8^. Its pivotal role in cell cycle regulation and tumor progression has made FOXM1 a major focus in cancer research^9^. The overexpression of FOXM1 has consistently been documented across various cancer types, including lung, breast, prostate, liver, and pancreatic cancers, as well as melanoma^10^-^15^. The upregulation of FOXM1 is positively correlated with increased proliferation, invasion, and metastasis of cancer cells and correlates significantly with tumor malignancy grading^16^, ^17^. FOXM1 plays a pivotal role in regulating the cell cycle, particularly during G1/S and G2/M transition phases^18^. It regulates cyclin expression and cell cycle regulatory gene expression, promoting S phase and mitosis progression, and facilitating cellular proliferation and growth. FOXM1 also regulates genes involved in cellular proliferation and apoptosis, including both pro-proliferative and anti-apoptotic genes^19^. The overexpression of FOXM1 promotes aberrant cellular proliferation and inhibits apoptosis, thereby promoting tumorigenesis. Moreover, FOXM1 plays a critical role in DNA damage repair and cellular stress response. Its rapid activation following DNA damage is intricately associated with cell survival and recovery^9^. Given FOXM1's pivotal role in tumor development, researchers are investigating its potential as a therapeutic target^20^. Inhibition of FOXM1 activity holds promise for effectively suppressing tumor cell proliferation and metastasis, emerging as a novel strategy for cancer treatment. Various FOXM1 inhibitors, including Thiostrepton, FDI-6, Siomycin A, RCM-1, and others, have been developed and are being investigated in the early stages of preclinical and clinical studies^21^. Further research is needed to evaluate their safety and efficacy. The critical role of FOXM1 in tumor development and cell biology has made it a prominent focus of cancer research. Understanding its mechanism of action is expected to provide new therapeutic strategies for melanoma treatment.

FOXM1 not only independently activates the transcription of tumor-related genes but also collaborates with other molecular pathways or transcription factors to enhance the phenotypic characteristics of tumor cells. The pathogenesis of melanoma involves multiple signaling pathways^22^-^24^. The PI3K/AKT pathway is among the most extensively studied and plays a crucial role in melanoma progression by promoting cell proliferation, migration, and invasion^25^. Targeting PI3K/AKT pathway inhibition thus represents a promising therapeutic approach for melanoma treatment. Mitogen-Activated Protein Kinase (MAPK) is a serine/threonine kinase involved in stress response and apoptosis. Three extensively described MAPK cascades are ERK (Extracellular Signal-Regulated Kinase), JNK (c-Jun N-terminal Kinase), and p38. The biological effects of MAPK signaling pathways manifest primarily through phosphorylation of downstream substrates, including transcription factors like c-Jun and c-Fos. ERK activation, primarily stimulated by mitogens, promotes cellular differentiation, proliferation, and survival^26^. In contrast, JNK and p38 are predominantly activated by oxidative stress and cytokines, eliciting inflammation and apoptosis^27^. The MAPK signaling pathway plays a pivotal role in various cellular processes, including proliferation, differentiation, apoptosis, and survival of melanoma cells^28^. Targeting the MAPK signaling pathway thus represents a pivotal therapeutic strategy for melanoma treatment^29^. Kim *et al.* demonstrated that quercetin effectively activates the JNK/P38 MAPK signaling pathway in A375SM melanoma cells, inducing apoptosis and inhibiting cancer cell viability^30^. Importantly, Yao *et al.* demonstrate that FOXO3 and FOXM1 forkhead box transcription factors play crucial roles in various cellular processes, including cell proliferation, differentiation, survival, senescence, DNA damage repair, and cell cycle control. They function downstream of the PI3K/AKT, Ras-ERK, and JNK/P38 MAPK signaling cascades^31^. The study conducted by Tian *et al.* revealed that miR-361-5p effectively targets FOXM1 through the PI3K/AKT/mTOR pathway, thereby inhibiting autophagy-induced chemotherapy resistance in gastric cancer cells^32^. Behren *et al.* demonstrated that FOXM1 acts as a downstream effector of the Ras-MKK3-p38 signaling pathway and regulates cellular invasion *in vitro*, as revealed by phenotyping-assisted transcriptome analysis^33^. Wang *et al.* demonstrated that inhibiting FOXM1 enhanced the chemosensitivity of docetaxel-resistant A549 cells to docetaxel by activating the JNK/ P38 MAPK mitochondrial pathway^34^. Collectively, these studies provide evidence that FOXM1 is positioned downstream of the PI3K/AKT and JNK/P38 MAPK signaling pathways to facilitate tumor progression. Consequently, targeting the transcriptional activity of FOXM1 or disrupting its interaction with other cancer-related pathways or transcription factors could represent a promising strategy for the development of novel anti-tumor therapeutics.

Lasalocid, a polyether ionophore antibiotic, is extensively employed as an animal feed additive, particularly in the husbandry of poultry and ruminants such as cattle and sheep^35^. However, in recent years, its potential as an antineoplastic agent has garnered significant attention. *In vitro* studies have unveiled the inhibitory effects of lasalocid on various cancer cell types, including breast, colon, and prostate cancers, among others^36^-^38^. The efficacy of lasalocid in inducing apoptosis and promoting autophagy in cancer cells has been demonstrated by studies. Lasalocid exerts its influence on the growth and division of cancer cells through the regulation of cell cycle proteins and associated signaling pathways. The study conducted by Kim *et al.* demonstrated that lasalocid effectively induces G0/G1 cell cycle arrest and apoptosis through mitochondrial and caspase-dependent pathways in prostate cancer^38^. Lasalocid promotes autophagy by enhancing autophagosome formation. Notably, reactive oxygen species (ROS) induction was associated with apoptosis and autophagy induced by lasalocid. Autophagy inhibition enhances lasalocid-induced apoptosis by increasing ROS production, suggesting autophagy plays a cytoprotective role resisting apoptosis. Although the anticancer activity of lasalocid has been studied, its effects on melanoma remain unreported. Whether lasalocid inhibits melanoma and the specific mechanism involved warrant further study.

The present study investigated the effects of lasalocid on human melanoma cells A375 and SK-MEL-28, demonstrating significant inhibitory effects on cell proliferation, migration, and invasion. Additionally, lasalocid induced cell cycle arrest in S phase and promoted apoptosis. Transcriptome sequencing and bioinformatics analysis revealed significant downregulation of FOXM1 expression upon lasalocid treatment compared to untreated cells. KEGG pathway analysis indicated significant enrichment of differentially expressed genes in PI3K/AKT and MAPK signaling pathways. Western blot analysis confirmed that lasalocid effectively inhibited FOXM1 expression through the PI3K/AKT and JNK/P38 MAPK pathways. The inhibitory effect of lasalocid on melanoma was confirmed through *in vivo* experiments, yielding consistent results. In summary, this study elucidated the molecular mechanism by which lasalocid inhibits melanoma by down-regulating FOXM1 via the PI3K/AKT and JNK/P38 MAPK pathways. These findings underscore the potential of lasalocid as a promising therapeutic agent for melanoma treatment.

## Materials and Methods

### Cell lines and materials

Human melanoma cell lines A375 and SK-MEL-28 were kindly provided by Procell (Procell, China). A375 cells were cultured in DMEM medium (GIBCO, USA) supplemented with 10% fetal bovine serum (FBS) (VivaCell, China) under a humidified atmosphere of 5% carbon dioxide at 37°C. Similarly, SK-MEL-28 cells were cultured in MEM medium (GIBCO, USA) supplemented with 10% FBS (VivaCell, China) under the same conditions. Lasalocid, SP600125, SB202190, and IGF1 proteins were purchased from MedChemExpress (MCE, USA). Lasalocid, SP600125, and SB202190 were diluted in dimethyl sulfoxide (DMSO) (Yamei, China) before use, and IGF1 Protein was diluted using the dilution that came with the package. The drug was dissolved in accordance with the recommended method provided by lasalocid instructions, followed by the sequential addition of 10% DMSO, 40% PEG300, 5% Tween-80, and 45% saline to obtain a clear and transparent solution during *in vivo* experiments.

### Cell viability

The viability of A375 and SK-MEL-28 cells was assessed using the CCK-8 kit (KeyGEN Biotechnology, China). Briefly, cells were seeded at a density of 1×10^3^ cells/well in 96-well plates and treated with various concentrations of lasalocid. Subsequently, 10% CCK-8 reagent was added to each well and incubated for 1 h at 37°C. The optical density (OD) value at 450 nm was measured using a microplate reader (Bio-Rad Laboratories, USA).

### Colony formation assay

The A375 and SK-MEL-28 cells (500 cells per well) were seeded in 6-well plates and treated with lasalocid. Upon the formation of visible colonies, consisting of more than 50 cells, the plates were fixed using 4% paraformaldehyde, stained with 0.5% crystal violet, and subsequently photographed.

### EdU incorporation assay

A375 and SK-MEL-28 cells were seeded in 24-well plates and treated with various concentrations of lasalocid. Cell proliferation was assessed using the kFluor488 Click-iT EdU imaging kit (KeyGEN Biotechnology, China) following the manufacturer's protocol. Briefly, cells were incubated with EdU (5 mmol/L) in the culture medium for 24 h. After fixation and permeabilization, cells were stained with the Click-iT reaction cocktail for 30 min and Hoechst 33342 at 5 mg/mL for 15 min, ensuring light protection from this step onwards. Subsequently, images were captured using a fluorescence microscope. Proliferating nuclei bound to EdU were labeled with green fluorescence.

### Cell cycle analysis with flow cytometry

The impact of drugs on the cell cycle was evaluated using a cell cycle kit (KeyGEN Biotechnology, China) following the manufacturer's instructions. A375 and SK-MEL-28 cells were initially seeded in 6-well plates at a density of 2×10^5^ cells/well and incubated for 24 h. Subsequently, the cells were treated with various concentrations of lasalocid. After a 24 h incubation period, cells were collected, washed thrice with cold PBS, and resuspended in 200 μL of PBS. Gradual addition of 70% ethanol was performed, and the samples were then stored overnight at -20°C. Subsequently, cells were stained with PI/RNase staining solution and analyzed for alterations in the cell cycle using flow cytometry (Agilent, USA).

### Apoptosis assay

To assess apoptosis, Annexin V and propidium iodide (PI) co-staining was utilized to distinguish between viable (Annexin V-, PI-), early apoptotic (Annexin V+, PI-), and late apoptotic (Annexin V+, PI+) cells. A375 and SK-MEL-28 cells treated with lasalocid were harvested in cold PBS and then washed twice with PBS. The Annexin V-FITC/PI apoptosis detection kit (KeyGEN Biotechnology, China) was used for apoptotic staining. Subsequently, flow cytometry analysis was conducted using an Agilent NovoCyte instrument (Agilent, USA).

### Migration and invasion assays

To assess cell migration, wound healing and Transwell assays were utilized. In the wound healing assay, A375 and SK-MEL-28 cells were seeded in 6-well plates at 2×10^5^ cells/well and cultured for 24 h. Then, cells were treated with various lasalocid concentrations. A linear wound was created with a 200 µL yellow microtube tip, and serum-free medium replaced complete medium. Wound healing was photographed with an inverted microscope. Wound closure was calculated by subtracting final from initial scratch area, dividing by initial area, and multiplying by 100%. For Transwell assays, A375 and SK-MEL-28 cells (10^4^ cells/well) were seeded in the upper chamber of a Transwell insert (Corning, USA) and cultured in serum-free medium. Complete medium with 10% FBS was added to the lower chamber. After 48 h, cells remaining on the upper surface were carefully removed with a cotton swab, while migrated cells on the lower surface were stained with 0.1% crystal violet. Quantification of migrated cells was done by random microscopy observation. The invasion assay was performed similarly to assess invasiveness of chamber cells, using a matrix gel-coated membrane (Corning, USA).

### Transcriptome sequencing and bioinformatics analysis

Transcriptome sequencing was performed by BGI Corporation. Following data acquisition, EXCEL and R language were utilized for organizing and analyzing differential gene data. Differentially expressed genes were imported into the Lianchuan biological platform (www.omicstudio.cn) to generate volcano plots illustrating gene expression differences. KEGG and GO enrichment analyses were performed on differentially expressed genes to elucidate molecular pathways and biological behaviors involved in lasalocid-mediated melanoma inhibition. The GEPIA2 database (gepia2.cancer-pku.cn) was employed to analyze target gene expression in melanoma tissues and patient prognosis impact.

### Western blot analysis

For protein analysis, cells underwent two washes with cold phosphate-buffered saline (PBS) (Biosharp, China) and were then lysed using high-strength RIPA buffer (Yamei, China). The resulting lysates were centrifuged at 13,000 g for 20 min at 4°C to remove insoluble components. The supernatant was mixed with SDS-PAGE loading buffer (Biyuntian, China) and heated to 100°C for five minutes before separation on 10% SDS-PAGE gels (Yamei, China). Proteins were subsequently transferred onto nitrocellulose (NC) membranes (Bio-Rad, USA) and blocked with Tris-buffered saline with Tween 20 (TBST) containing 5% skim milk powder for two hours at room temperature. Primary antibodies targeting cyclinA2, CDK2, MMP2, MMP9, Bcl2, Bax, PI3K, phosphorylated PI3K, AKT, phosphorylated AKT, p38, phosphorylated P38, JNK, phosphorylated JNK, and GAPDH (Sanying, China) were incubated overnight at 4°C. Following three washes with TBST, the membranes were incubated with goat anti-rabbit IgG and HRP-linked antibodies for one hour at room temperature. Bands were visualized using an ECL detection kit according to the manufacturer's instructions, with GAPDH serving as an internal reference for protein quantification. Protein concentration was determined through film development.

### RNA extraction and real-time quantitative PCR (qPCR) assay

Total RNA was extracted from A375 and SK-MEL-28 cells using a Novozam column extraction kit, following the manufacturer's instructions (Novozam Biotech, China). Subsequently, reverse transcription of RNA was performed, followed by real-time PCR analysis using ChamQ SYBR Color qPCR Master Mix (Novozam Biotech, China). The thermal cycling conditions were as follows: initial denaturation at 94°C for 30 seconds; amplification consisting of 35 cycles with denaturation at 94°C for 30 seconds, annealing at 55°C for 30 seconds, extension at 72°C for 30 seconds; final extension step at 72°C for 10 minutes; and holding temperature set to 4°C. Expression levels were normalized to GAPDH and quantified using the 2^(-ΔΔ Ct) method. The primer sequences (5'-3') employed in this study are provided below:

FOXM1-forward: ATACGTGGATTGAGGACCACT; FOXM1-reverse: TCCAATGTCAAGTAGCGGTTG; CyclinA2-forward: CGCTGGCGGTACTGAAGTC; CyclinA2-reverse: GAGGAACGGTGACATGCTCAT; CDK2-forward: CCAGGAGTTACTTCTATGCCTGA; CDK2-reverse: TTCATCCAGGGGAGGTACAAC; MMP2-forward: CCCACTGCGGTTTTCTCGAAT; MMP2-reverse: CAAAGGGGTATCCATCGCCAT; MMP9-forward: GGGACGCAGACATCGTCATC; MMP9-reverse: TCGTCATCGTCGAAATGGGC; BCL2-forward: GGTGGGGTCATGTGTGTGG; BCL2-reverse: CGGTTCAGGTACTCAGTCATCC; BAX-forward: CCCGAGAGGTCTTTTTCCGAG; BAX-reverse: CCAGCCCATGATGGTTCTGAT; GAPDH-forward: GGAGTCCACTGGCGTCTTCA; GAPDH-reverse: GTCATGAGTCCTTCCACGATACC.

### Immunofluorescence analysis

The melanoma cells were treated with lasalocid for 24 h. Following treatment, the cells were fixed with 4% paraformaldehyde (Biosharp, China) for 15 min. Subsequently, the cells were permeabilized using 0.5% Triton X-100 (Solarbio, China) for 10 min. Afterwards, the melanoma cells were blocked with BSA blocking solution (Solarbio, China) for 30 min and then incubated overnight at 4°C with anti-FOXM1 antibody (Sanying, China). The cells underwent three washes with PBS and were then incubated in a chamber with AlexaFluor488-labeled goat anti-rabbit IgG antibody (Sanying, China) for 2 h. Subsequently, the nuclei were stained at room temperature for 10 min using Hoechst33342 (UElandy, China). Finally, immunofluorescence observations were examined under an immunofluorescence microscope (Leica, Germany).

### Animal experiment

Male BALB/c nude mice (4-6 weeks old, approximately 20 g) were obtained from Beijing Speford Biotechnology Co., Ltd (Beijing, China) (license number: SCXK(Beijing)2019-0010) and were housed in specific pathogen-free, climate-controlled facilities with a maximum of five animals per cage. The mice were provided ad libitum access to food and water. All animal experiments were conducted following the approved guidelines of the Ethics Committee of North Sichuan Medical College (Ethical Review number: 2023076). Sixteen male BALB/c nude mice were included in the study, and subcutaneous implantation of A375 cells (3×10^6^ cells in 100 µL PBS) was performed on the right flank of each mouse. When the tumors reached a volume of approximately 200 mm^3^, the mice were randomly divided into two groups for intraperitoneal injection treatment. The groups consisted of: (1) a control Saline group (200 µL Saline/mouse), and (2) a lasalocid group (7.5 mg/kg). Tumor volume was measured using calipers, and the body weight of the mice was recorded every other day. Tumor volume (V) was calculated using the formula (A×a^2^)/2, where A and a represent the long and short diameters of the tumor, respectively. Statistical differences between groups were analyzed using one-way analysis of variance (ANOVA). On the 14th day after treatment initiation, euthanasia was performed on all mice. Tumor and visceral tissues from each mouse were fixed in 4% paraformaldehyde and embedded in paraffin for subsequent histological analysis.

### Immunohistochemistry analysis

The paraffin-embedded tumor blocks were sectioned and subjected to immunohistochemistry using the high-sensitivity SP (streptavidin-perosidase) immunohistochemical kit (Maixin, China). Briefly, sections were deparaffinized with xylene and rehydrated through graded ethanol. Antigen retrieval was performed by heating sections in 10 mM citrate buffer (pH 6.0). Endogenous peroxidase activity was blocked with 50 μL hydrogen peroxide solution (reagent A) for 10 min, followed by blocking with 50 μL blocking serum (reagent B) for 60 min at room temperature. Sections were then incubated overnight at 4°C with polyclonal rabbit antibodies against Ki67, FOXM1, MMP2, and MMP9 (Sanying, China) at 1:500 dilution. Subsequently, sections were incubated with biotinylated goat anti-rabbit secondary antibody (reagent C) for 20 min at room temperature. Following this, sections were treated with avidin and biotinylated horseradish peroxidase (reagent D) for another 20 min. Immunoreactivity was visualized using an ABC kit (Maixin, China) with diaminobenzidine as substrate. Finally, selected sections were counterstained with hematoxylin and examined under a microscope (Leica, Germany).

### Hematoxylin-eosin (HE) staining

Hematoxylin-eosin (HE) staining was performed using the HE staining kit protocol (Solarbio, China). Briefly, sections (2μm thick) were stained with hematoxylin for 1 min and then subjected to acidic liquid alcohol differentiation for 30 seconds. After a 50-second eosin staining period, the cells were dehydrated with ethanol (95%, 100%) and finally cleared with xylene and fixed. The results were analyzed under a microscope from Leica (Leica, Germany).

### TUNEL staining

Apoptosis in tumor tissues was assessed using a TUNEL staining kit (Yeasen, China). Three randomly selected microscopic fields per slide were examined under a microscope (Leica, Germany).

### Statistical analyses

Statistics are presented as mean ± standard deviation (mean±SD). T-tests and analysis of variance (ANOVA) were performed using Excel and GraphPad Prism 9 software. One-way ANOVA was performed on more than two groups of samples, with multiple comparisons used to assess inter-group differences. All experiments were performed in at least three independent replicates. The following symbols were used to indicate statistically significant differences: no significance, *P* > 0.05; significance, *P* < 0.05.

## Results

### Lasalocid inhibits the proliferation of human melanoma cells

**Figure 1:**
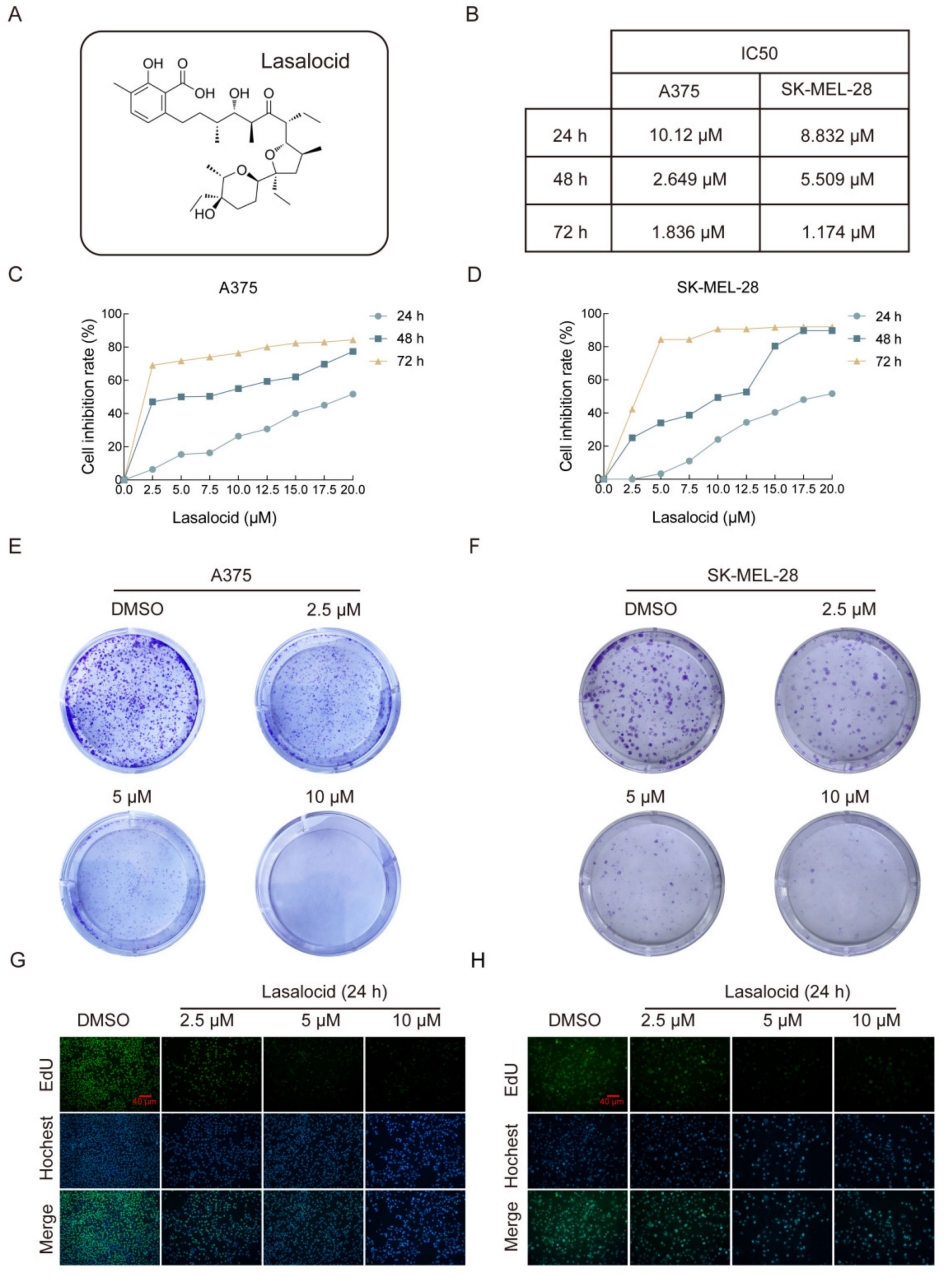
Lasalocid inhibits the proliferation of human melanoma cells. (A) Chemical structure of lasalocid. (B) IC50 of A375 and SK-MEL-28 cells in three time periods of 24 h, 48 h, and 72 h. (C,D) The inhibitory rates of lasalocid on A375 and SK-MEL-28 cells at different times and concentrations were detected by CCK-8 assay. (E,F) Formation of clones was used to detect the ability of lasalocid to inhibit the proliferation of A375 cells. (G,H) EdU assay was used to detect the ability of lasalocid to inhibit the proliferation of A375 and SK-MEL-28 cells. (Data are mean±SD *P<0.05, **P <0.01, ***P <0.001, ****P<0.0001).

The chemical structure of lasalocid is illustrated in Figure 1A. To investigate the anti-cancer activity of lasalocid in melanoma cells, we initially evaluated the impact of varying concentrations (2.5, 5, 10, 12.5, 15, 17.5, and 20 μM) of lasalocid on the proliferation of two distinct melanoma cell lines (A375 and SK-MEL-28). The results are depicted in (Fig. 1B-D), revealing a dose-dependent and time-dependent reduction in proliferation for both A375 and SK-MEL-28 cells following treatment with lasalocid for 24 h, 48 h, or 72 h. The semi-inhibitory concentration of A375 cells and SK-MEL-28 cells treated with lasalocid for 24 h was 10.12 μM and 8.832 μM, respectively. Subsequently, treatment with lasalocid for 48 h resulted in semi-inhibitory concentration values of 2.649 μM for A375 cells and 5.509 μM for SK-MEL-28 cells. Finally, a 72 h treatment with lasalocid led to semi-inhibitory concentration values of 1.836 μM for A375 cells and 1.174 μM for SK-MEL-28 cells. Therefore, lasalocid at concentrations of 2.5 μM, 5 μM, and 10 μM was selected for subsequent experiments. The inhibitory effect of lasalocid on the proliferation of melanoma cells was further verified by colony formation and EdU experiments (Fig. 1E-H). In conclusion, CCK-8 assay, Formation of clones, and EdU assay consistently demonstrated that lasalocid inhibited the proliferation ability of melanoma cells in a concentration-dependent manner.

### Lasalocid exhibits inhibitory effects on the S phase of melanoma cell cycle and induces cellular apoptosis

**Figure 2:**
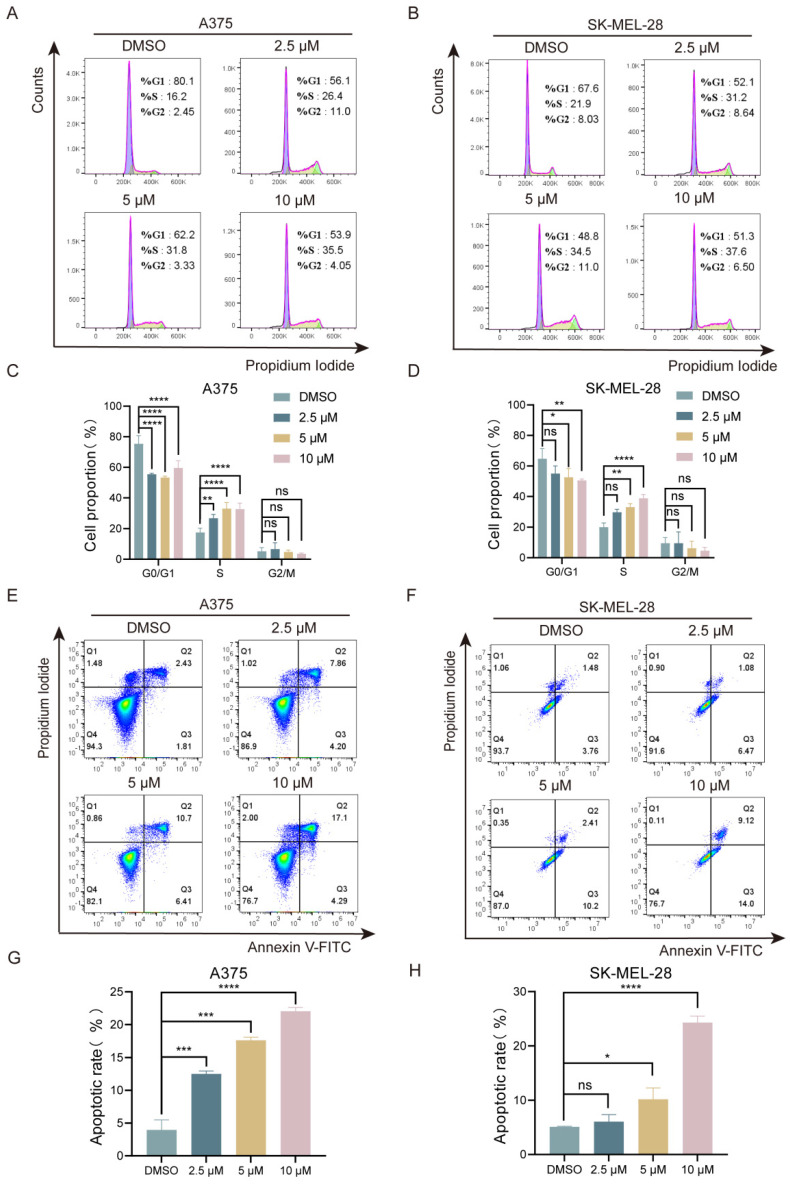
Lasalocid inhibits melanoma cell cycle and induces apoptosis. (A,B) Flow cytometry was used to detect the effect of different concentrations of lasalocid on the cell cycle distribution of melanoma A375 and SK-MEL-28 cells. (C,D) Statistical plots of melanoma cell cycle in groups treated with different concentrations of lasalocid. (E,F) Changes in apoptosis levels of melanoma cells treated with different concentrations of lasalocid. (G,H) Statistical plot of the percentage of apoptotic cells. (Data are mean±SD *P<0.05, **P <0.01, ***P <0.001, ****P<0.0001).

Next, we investigated the effects of lasalocid on cell cycle and apoptosis in melanoma cells. The A375 and SK-MEL-28 cells were exposed to various concentrations of lasalocid for a duration of 24 h. The distribution of cells in different phases of the cell cycle was then analyzed using flow cytometry and PI staining. The results showed that the percentage of S-phase cells in A375 cells increased from 16.2% to 26.4%, 31.8%, and 35.5% after treatment with 0, 2.5, 5, and 10 µM lasalocid, respectively (Fig. 2A,C). Similarly, in SK-MEL-28 cells, the percentage of S-phase cells increased from 21.9% to 31.2%, 34.5%, and 37.6% (Fig. 2B,D). Moreover, flow cytometry analysis revealed a significant increase in apoptosis in both A375 cells (4.24%, 12.06%, 17.11%, and 21.39%) and SK-MEL-28 cells (5.24%, 7.55%, 12.61%, and 23.12%) after treatment with different concentrations of lasalocid for 24 h (Fig. 2 E-H). These findings suggest that lasalocid induces apoptosis by inhibiting the S phase of cells and exhibits an anti-proliferative effect on melanoma cells *in vitro*.

### Lasalocid inhibits the migration and invasion of melanoma cells

**Figure 3:**
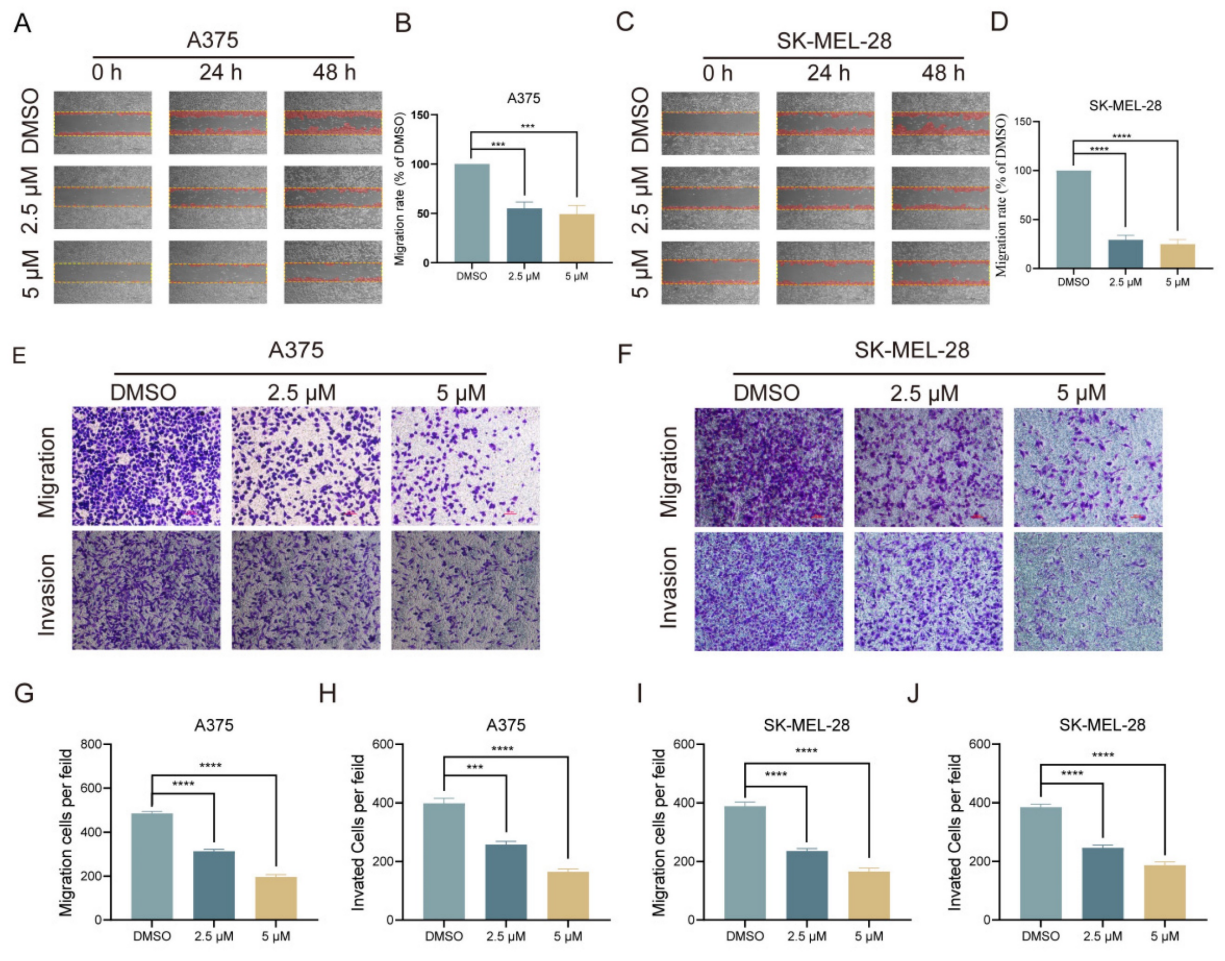
Lasalocid inhibits the migration and invasion of melanoma cells. (A,C) Scratch healing of melanoma A375 and SK-MEL-28 cells treated with different concentrations of lasalocid. (B,D) Statistical plot of the percentage of scratched wound area. (E,F) Transwell was used to detect the migration and invasion of melanoma cells treated with different concentrations of lasalocid. (G,J) Statistical plot of the number of migrating and invading melanoma cells. (Data are mean±SD *P<0.05, **P <0.01, ***P <0.001, ****P<0.0001).

Melanoma, a prevalent and highly aggressive malignancy worldwide, has prompted our investigation into the impact of lasalocid on melanoma cell migration and invasion. Initially, a scratch healing assay was employed to assess the inhibitory potential of lasalocid on melanoma cell migration *in vitro*. Our experimental findings revealed a significant reduction in the migratory capacity of melanoma cells after 48 h of lasalocid treatment compared to the control group (Fig. 3A-D). After the scratch healing assay, a transwell assay was utilized to further verify the migration and invasion capabilities of melanoma cells in response to lasalocid treatment. The results exhibited a noteworthy decrease in both migration and invasion potential of melanoma cells treated with lasalocid for 48 h, in comparison to the control group (Fig. 3E-J). In conclusion, lasalocid effectively impedes the migratory and invasive capabilities of melanoma cells.

### FOXM1 is a key gene in the inhibition of melanoma by lasalocid

**Figure 4:**
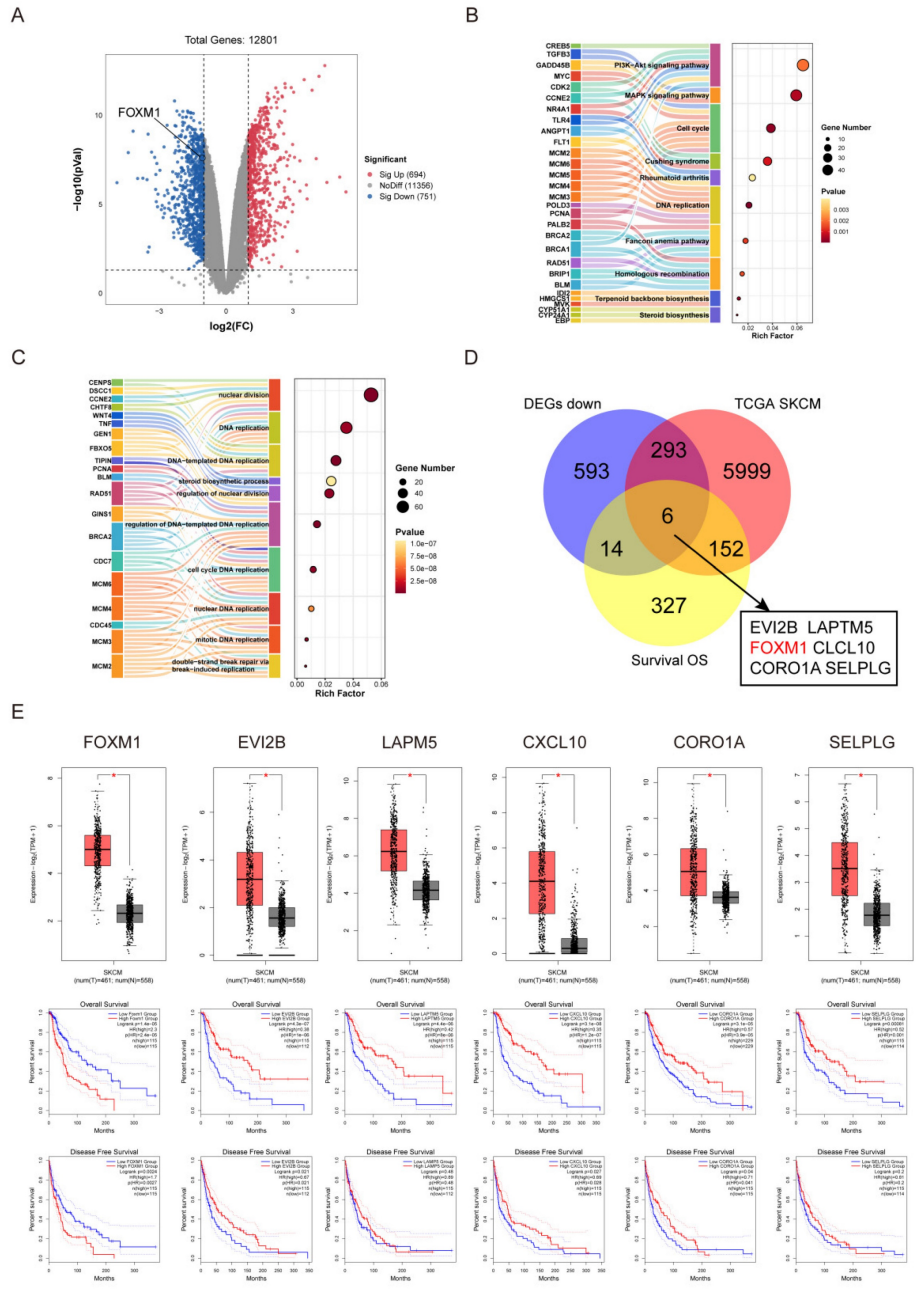
FOXM1 is a key gene in the inhibition of melanoma by lasalocid (A) Volcano plot of differential genes. (B) KEGG pathway analysis of differentially expressed genes. (C) GO functional enrichment analysis of differentially expressed genes. (D) Venn diagram was used to screen the key genes of lasalocid inhibiting melanoma. (E) GEPIA2 online analysis platform was used to verify the candidate genes.

We sequenced the genes of the control and lasalocid treated groups, and represented the differentially expressed genes between these groups using a volcano plot. Red dots indicate up-regulated genes, blue dots indicate down-regulated genes, and grey dots represent genes with no significant differential expression (*P* < 0.05, |log_2_FC| ≥ 1.0) (Fig. 4A). The genes identified as differentially expressed through RNA sequencing were subjected to Gene Ontology (GO) and Kyoto Encyclopedia of Genes and Genomes (KEGG) pathway analyses. The KEGG pathway analysis revealed that these genes were predominantly associated with key signaling pathways, including the PI3K/AKT signaling pathway, MAPK signaling pathway, Cell cycle, Cushing syndrome, Fanconi anemia pathway, Homologous recombination, Terpenoid backbone biosynthesis, Circadian rhythm, and Steroid biosynthesis (Fig. 4B). On the other hand, the GO analysis indicated significant differences in gene biological processes, encompassing nuclear division, DNA replication (templated and non-templated), steroid biosynthetic process, regulation of nuclear division, cell cycle DNA replication (nuclear and mitotic), nuclear DNA replication, mitotic DNA replication, and double-strand break repair via break-induced replication (Fig. 4C). In order to screen the key genes of lasalocid inhibiting melanoma, we combined the down-regulated genes treat with lasalocid (DEGs down), the genes highly expressed in melanoma in the TCGA database (TCGA SKCM), and the genes related to the survival of melanoma patients in the TCGA database (Survival OS), and we identified six candidate genes: EVIEVI2B, LAPTM5, FOXM1, CXCL10, CORO1A and SELPLG (Fig. 4D). The online analysis platform GEPIA2 was used to further verify these candidate genes, and it was found that all six genes were highly expressed in tumor tissues, but only the high expression of FOXM1 was associated with poor prognosis (Fig. 4E). Collectively, these findings suggested that the transcription factor FOXM1 may exert a pivotal role in mediating the impact of lasalocid on melanoma cell proliferation and migration.

### Lasalocid inhibits the proliferation, migration and invasion of melanoma cells by down-regulating FOXM1

**Figure 5:**
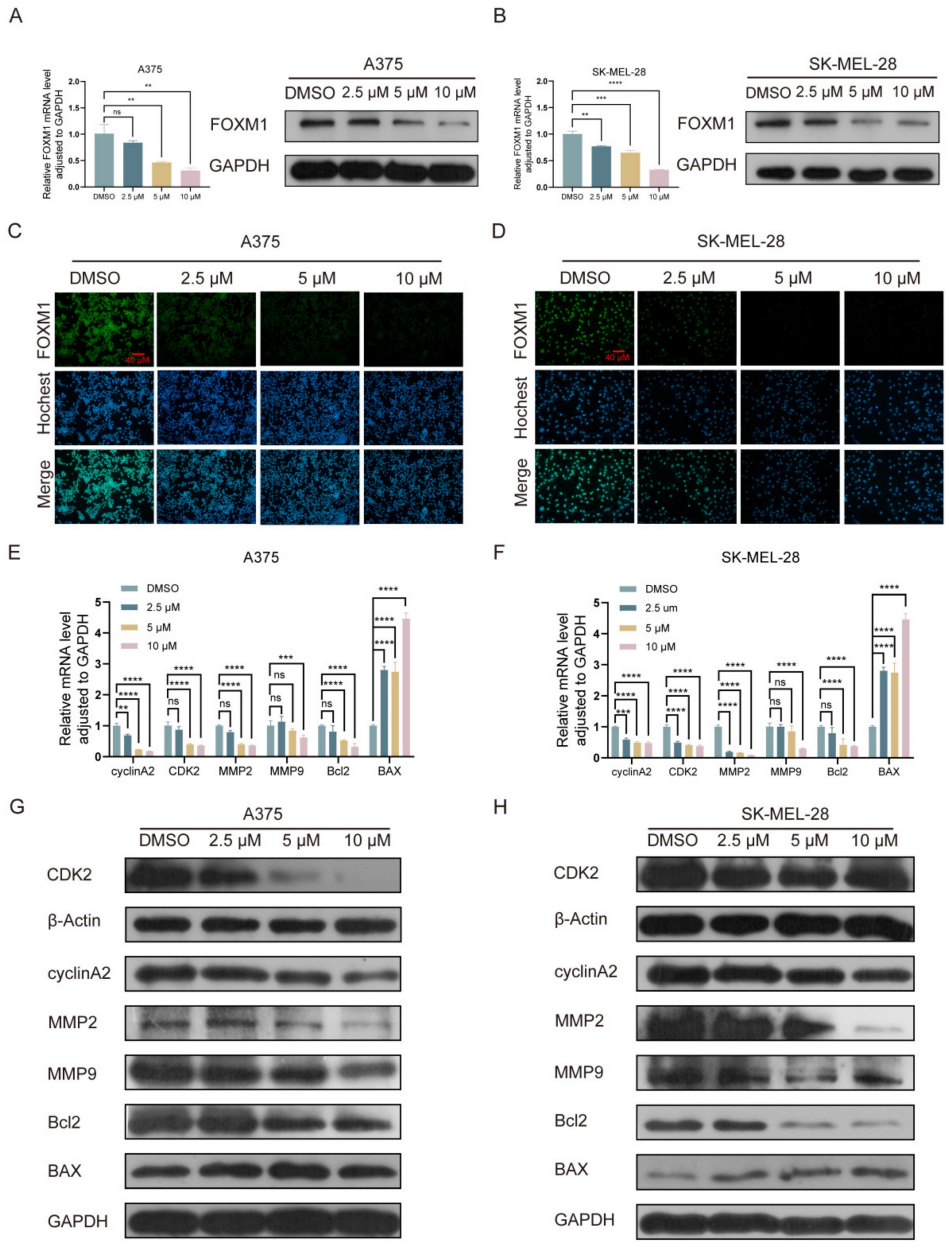
Lasalocid inhibits the proliferation, migration and invasion of melanoma cells by down-regulating FOXM1. (A,B) The inhibition of FOXM1 mRNA and protein expression by lasalocid in human melanoma cells was assessed using real-time PCR and Western blot techniques. (C,D) Immunofluorescence assay was used to detect the inhibition of FOXM1 protein expression by lasalocid. (E,F) The mRNA expression of FOXM1-regulated genes in human melanoma cells treated with lasalocid for 24 h was quantitatively detected using real-time quantitative PCR. (G,H) Western blot was used to detect the expression of FOXM1-regulated proteins in human melanoma cells treated with lasalocid for 24 h. (Data are mean±SD *P<0.05, **P <0.01, ***P <0.001, ****P<0.0001).

The expression of FOXM1 mRNA and protein was significantly downregulated in A375 and SK-MEL-28 cells following a 24 h treatment with lasalocid (Fig. 5 A-D). The significance of FOXM1 in cell mitosis primarily lies in its regulation of the G1 to S phase and G2 to M phase transitions, with aberrant expression potentially resulting in cell cycle arrest and chromosomal missegregation within tumor cells^39^, ^40^. Wang and Gabriel T. M. Lok *et al.* demonstrated that the down-regulation of FOXM1 resulted in the inhibition of matrix metalloproteinases (MMPs), including MMP2 and MMP9, thereby suppressing tumor cell migration and invasion^40^, ^41^. Moreover, Qin *et al.* demonstrated that the down-regulation of FOXM1 resulted in the up-regulation of BAX protein and down-regulation of Bcl2 protein, leading to a significant induction of apoptosis in esophageal cancer cells^42^. We have demonstrated the inhibitory effects of lasalocid on the proliferation, migration and invasion of melanoma cells. Additionally, we observed that lasalocid effectively arrested cell cycle progression in the S phase and induced apoptosis. To further elucidate the underlying mechanism of action, we investigated FOXM1-interacting proteins associated with cell cycle regulation, migration dynamics, invasion potential, and apoptotic pathways. The expression of cyclin A2 and CDK2, as well as MMP2 and MMP9, was significantly suppressed in A375 and SK-MEL-28 cells following 24 h of lasalocid treatment, as determined by real-time PCR and Western blot analysis. Additionally, the level of the apoptosis inhibitor protein Bcl2 was reduced while the expression of the apoptosis-promoting protein BAX was increased (Fig. 5 E-H). Collectively, these studies have demonstrated that lasalocid exerts regulatory effects on the expression of proteins involved in cell cycle progression, migration, invasion, and apoptosis by modulating downstream targets of FOXM1 transcriptional activity.

### Lasalocid inhibits melanoma cell proliferation by down-regulating FOXM1 by inhibiting the PI3K/AKT pathway

**Figure 6:**
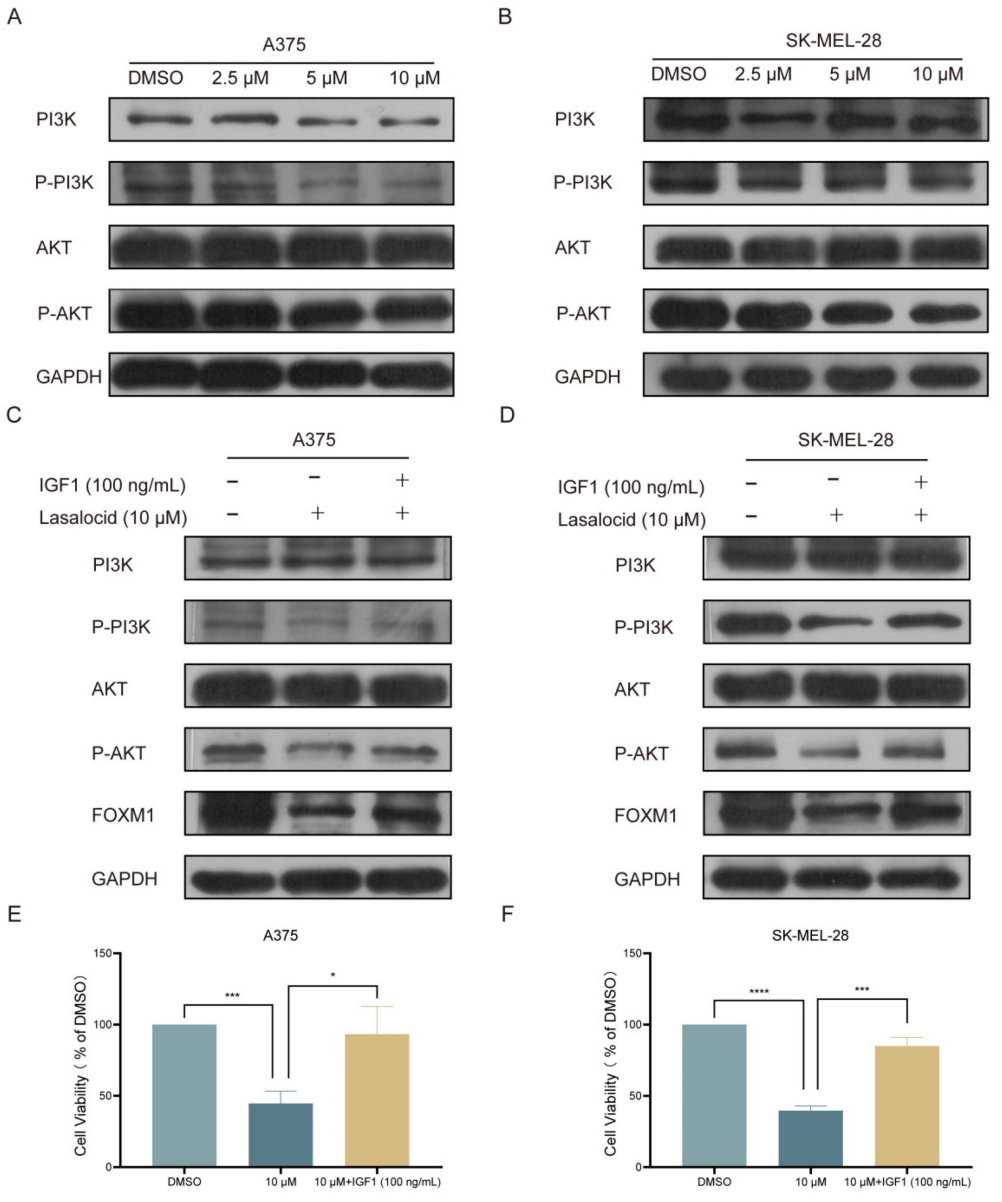
FOXM1 was down-regulated by lasalocid through inhibition of PI3K/AKT pathway. (A,B) The protein expression levels of PI3K, AKT, P-PI3K, and P-AKT in melanoma cells were assessed after 24 h of lasalocid treatment using Western blot analysis. (C,D) Western blot analysis was employed to assess the expression levels of PI3K, AKT, P-PI3K, P-AKT and FOXM1 following activation of the PI3K/AKT pathway by IGF1 (100 ng/mL, 24 h). (E,F) Statistical diagram of the proliferation ability of melanoma cells significantly increased by IGF1 detected by CCK-8. (Data are mean±SD *P<0.05, **P <0.01, ***P <0.001, ****P<0.0001).

The PI3K/AKT pathway represents a pivotal cellular signaling cascade implicated in the regulation of diverse biological processes, encompassing cell proliferation, survival, differentiation, and metabolism. This intricate pathway encompasses several crucial constituents comprising phosphatidylinositol 3-kinase (PI3K), phosphatidylinositol dependent kinase (PDK1), protein kinase B (PKB or AKT), and the mTOR complex. In the activated state, external signals (such as growth factors and cytokines) initiate PI3K activation via receptors, leading to the conversion of phosphatidylinositol diphosphate (PIP2) into phosphatidylinositol triphosphate (PIP3). Subsequently, PIP3 activates PDK1 and AKT, thereby initiating a cascade of downstream signaling pathways that modulate cellular functions^43^. Western blot analysis was conducted to validate the expression of the PI3K/AKT pathway in melanoma cells following 24 h of lasalocid treatment. The findings demonstrated a significant inhibition of P-PI3K and P-AKT expression by lasalocid, while total PI3K and AKT expression remained unaffected (Fig. 6A,B). Subsequent investigations revealed that insulin growth factor-1 (IGF1), a peptide capable of activating the PI3K/AKT pathway, significantly up-regulated the expression levels of phosphorylated PI3K and AKT (P-PI3K and P-AKT, respectively), while exerting no significant impact on the overall expression of PI3K and AKT. Concurrently, activation of the PI3K/AKT pathway by IGF1 also led to a substantial up-regulation in FOXM1 protein expression (Fig. 6C,D). Moreover, the activation of the PI3K/AKT pathway by IGF1 significantly enhanced the proliferation of A375 and SK-MEL-28 cells (Fig. 6E,F). Collectively, these findings suggest that lasalocid may impede melanoma cell proliferation by downregulating FOXM1 via modulation of the PI3K/AKT signaling pathway.

### Lasalocid inhibits melanoma cell proliferation by down-regulating FOXM1 via activating JNK/P38 MAPK pathway

**Figure 7:**
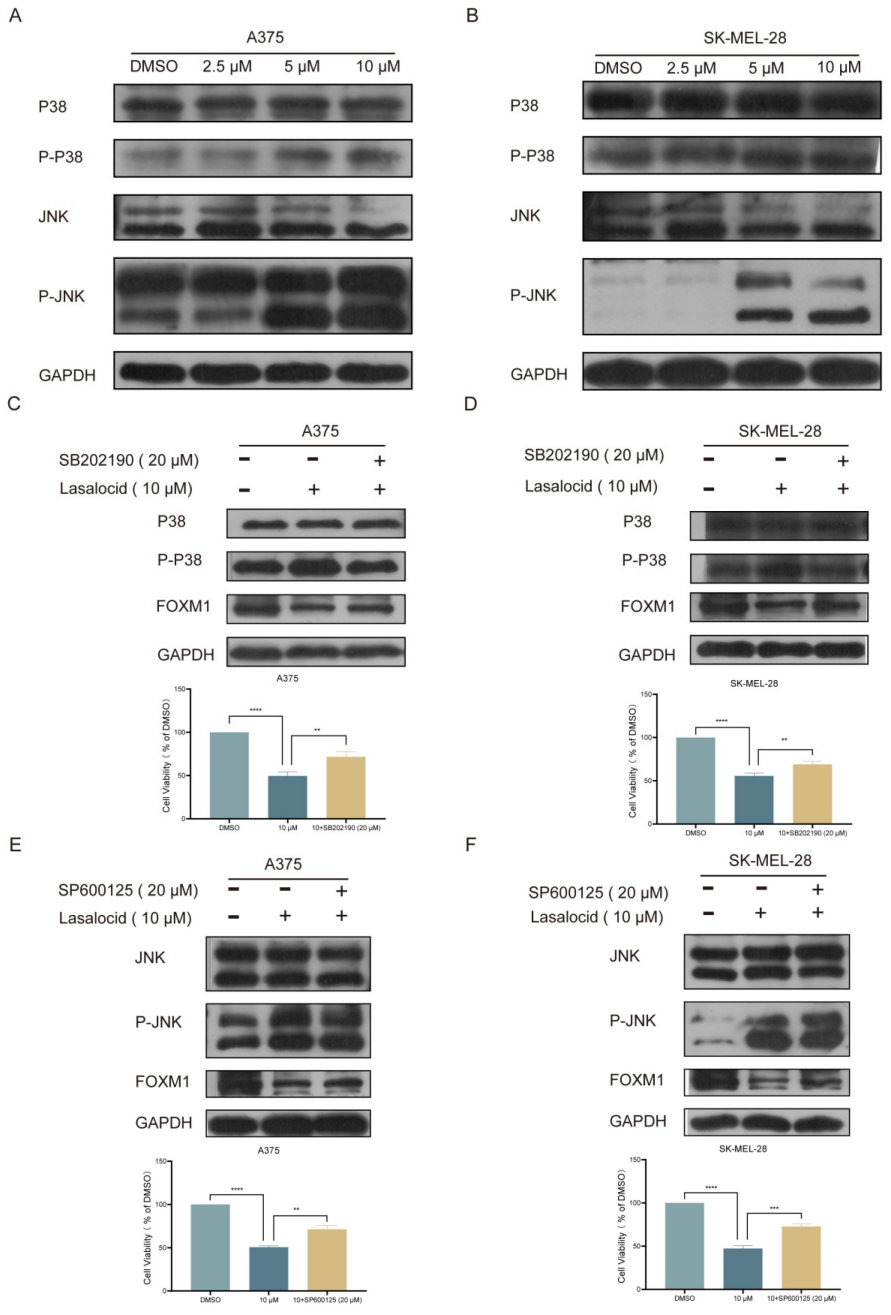
Lasalocid inhibits the proliferation of melanoma cells by activating JNK/P38 MAPK pathway and down-regulating FOXM1. (A,B) The protein expression levels of JNK, P-JNK, P38 and P-P38 in melanoma cells treated with lasalocid for 24 h were detected by Western blot. (C,D) The expression of P38, P-P38 and FOXM1 proteins and the proliferation ability of melanoma cells after inhibiting the P38 MAPK pathway by SB202190 (20 μM, 24 h). (E,F) The expression of JNK, P-JNK and FOXM1 proteins and the proliferation ability of melanoma cells after inhibiting JNK MAPK pathway by SP600125 (20 μM, 24 h). (Data are mean±SD *P<0.05, **P <0.01, ***P <0.001, ****P<0.0001.

Mitogen-Activated Protein Kinase (MAPK) is a well-established member of the serine/threonine kinase family, playing a pivotal role in stress response and apoptosis. Currently, three extensively characterized MAPK cascades have been identified: ERK (Extracellular Signal-Regulated Kinase), JNK (c-Jun N-terminal Kinase), and P38^28^. The biological effects of MAPK signaling pathways are predominantly mediated through the phosphorylation of downstream substrates, including transcription factors such as c-Jun and c-Fos. ERK is primarily activated by mitogens, thereby orchestrating cellular differentiation, proliferation, and survival. In contrast, JNK and P38 are predominantly activated by oxidative stress and cytokines, eliciting inflammation and apoptosis^27^. Kim *et al.* demonstrated that the activation of the JNK/P38 MAPK pathway exerts inhibitory effects on melanoma cell proliferation and induces apoptosis. Western blot analysis was conducted to validate the expression of the JNK/P38 MAPK pathway in human melanoma cells following 24 h of lasalocid treatment. The findings demonstrated a significant induction of P-P38 and P-JNK expression by lasalocid, while no impact on total P38 and JNK expression was observed (Fig. 7A,B). SB202190, a P38 MAPK pathway inhibitor, was used to inhibit the P38 MAPK pathway. The results showed that P-P38 was significantly down-regulated, while total P38 was not changed (Fig. 7C,D). SP600125, an inhibitor of the JNK MAPK pathway, was also used and showed a significant down-regulation of P-JNK, while total JNK was unchanged (Fig. 7 E,F). Notably, FOXM1 expression was significantly up-regulated after inhibition of the P38/ JNK MAPK pathway (Fig. 7C-F). In addition, the proliferation of A375 and SK-MEL-28 cells was significantly increased after JNK/P38 MAPK pathway inhibition (Fig. 7C-F). Collectively, the findings indicate that lasalocid exhibits potential as a suppressor of melanoma cell proliferation through the down-regulation of FOXM1 via activation of the JNK/P38 MAPK pathway.

### Lasalocid inhibits melanoma cell proliferation *in vivo*

**Figure 8:**
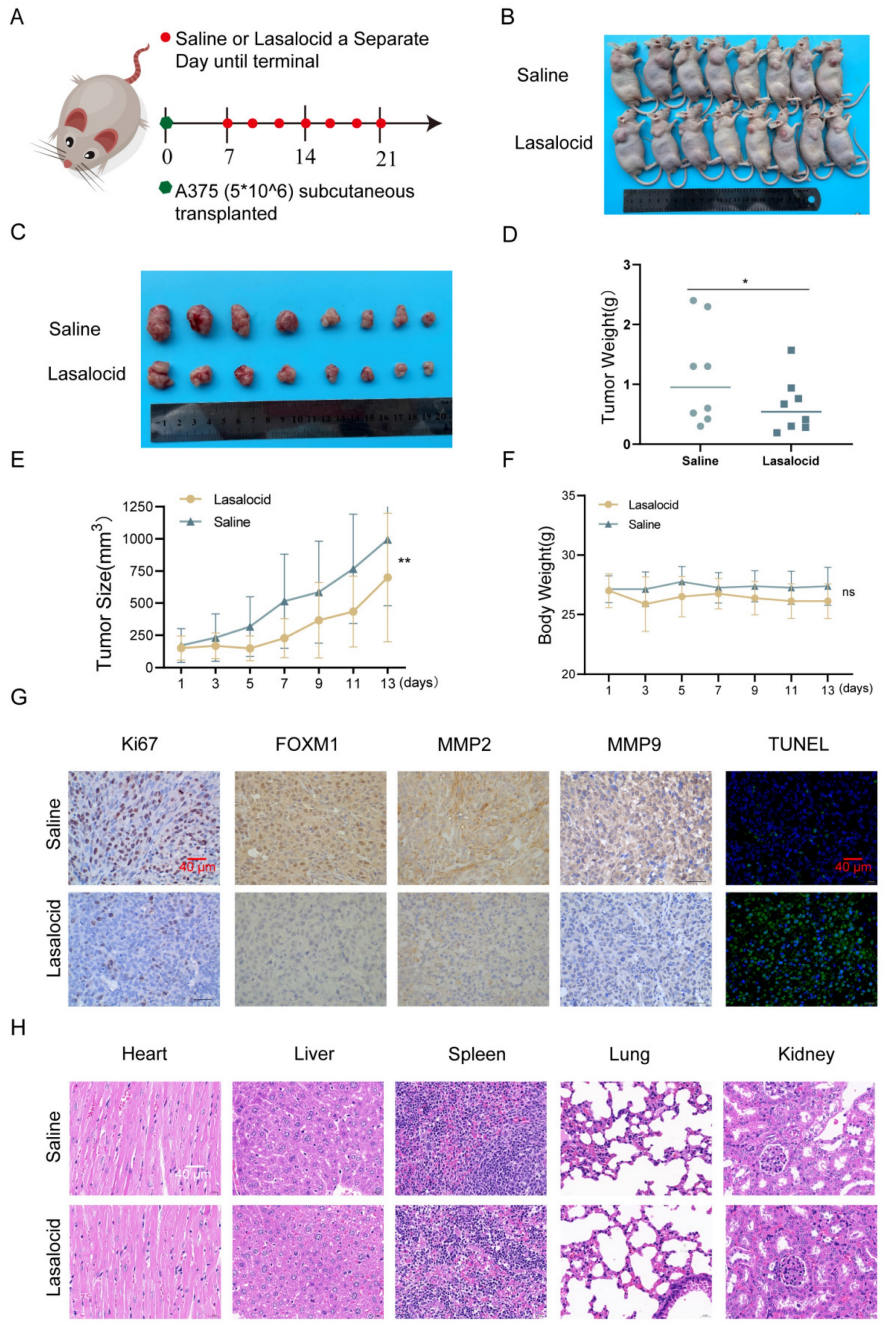
Lasalocid inhibits melanoma cell proliferation *in vivo* (A) Schematic representation of *in vivo* experiments. (B) Photos of nude mice after tumor formation. (C) Images of the stripped tumor. (D) Statistical plot of tumor mass. (E) Statistical plot of mouse weight. (F) Statistical plot of mouse weight. (G) Immunohistochemistry was used to detect the expression of FOXM1, MPP2, MMP9 and Ki67 protein in the tumors. TUNEL staining was used to detect the apoptosis of tumors *in vivo*. (H) Heart, liver, spleen, lung and kidney tissues were examined by HE to assess the systemic toxicity of lasalocid. (Data are mean±SD *P<0.05, **P <0.01).

A375 cells were subcutaneously implanted into BALB/c nude mice, and once palpable tumors developed, the mice received intraperitoneal injections of lasalocid (7.5 mg/kg) or an equivalent volume of saline every other day for two weeks. The results demonstrated that lasalocid significantly suppressed tumor proliferation without affecting the body weight of nude mice (Fig. 8A-F). Xenograft tumor indicators were quantitatively assessed by staining tumor sections for protein expression. The results revealed comparatively lower levels of FOXM1, MMP2, MMP9, and Ki67 staining in the lasalocid group compared to the control group (Fig. 8G). Additionally, TUNEL staining showed significantly enhanced fluorescence intensity in the lasalocid group compared with the control group (Fig. 8G), indicating increased apoptosis. Thus, the data suggest the efficacy of lasalocid against melanoma *in vivo*. To evaluate the safety profile of lasalocid in terms of tumor suppression, heart, liver, spleen, lung, and kidney tissues were extracted from mice, fixed, and stained with hematoxylin-eosin (H&E). Microscopic analysis of organ tissue revealed no apparent systemic toxicity following treatment with lasalocid (Fig. 8H). Consequently, it can be concluded that lasalocid exhibits favorable tolerability in mice while imparting a certain level of biosafety. In conclusion, lasalocid effectively suppressed melanoma cell proliferation *in vivo* while maintaining relative biosafety.

## Discussion

Melanoma is a skin cancer with high incidence, poor prognosis and complex pathogenesis^44^. Despite decades of development, the overall prognosis of melanoma remains dismal, and existing therapeutic drugs have failed to significantly enhance patient survival. Particularly in the realm of cytotoxic agents, despite numerous research endeavors, their efficacy remains unsatisfactory^45^. Lasalocid, a polyether antibiotic, is widely employed as a feed additive in poultry and livestock to effectively prevent and treat specific parasitic diseases in these animals^46^. The latest research findings have revealed that lasalocid exhibits not only significant agricultural utility, but also demonstrates promising potential as an antitumor agent in the field of medicine^47^. Numerous studies have demonstrated the inhibitory effects of lasalocid on tumor cell proliferation, growth, and cancer progression through induction of apoptosis in tumor cells. However, the precise mechanism underlying its anti-melanoma activity remains elusive. The viability of A375 and SK-MEL-28 melanoma cells was systematically assessed using the CCK-8 assay. Lasalocid effectively suppressed the viability of both melanoma cell lines in a time- and dose-dependent manner. To further investigate the inhibitory effect of lasalocid on melanoma cell proliferation, we performed colony formation and EdU incorporation assays. Lasalocid treatment significantly reduced both colony formation ability of melanoma cells and the number of EdU-positive cells, indicating effective suppression of cell proliferation. Subsequently, we conducted a systematic examination of the impact of lasalocid on cell cycle progression and apoptosis in melanoma cells using flow cytometry. Lasalocid significantly arrested the melanoma cell cycle at the S phase and induced apoptosis. Finally, we validated the impact of lasalocid on melanoma cell migration and invasion through cell scratch and Transwell migration assays. Lasalocid effectively suppressed melanoma cell migration and invasion. Overall, our study elucidated the regulatory effects of lasalocid on melanoma cell proliferation, cell cycle progression, and apoptosis, while also confirming its potential as a therapeutic agent for inhibiting melanoma cell migration and invasion.

FOXM1 is a typical proliferation related transcription factor, belonging to the Forkhead box (FOX) transcription factor family^7^. In terms of gene function, FOXM1 is a pivotal protein that governs the regulation of cell cycle progression and cellular proliferation^39^. FOXM1 plays a pivotal role in orchestrating the transcriptional regulation of diverse genes implicated in cell cycle progression, thereby exerting precise control over DNA replication and mitotic processes. Inhibition of FOXM1 effectively abrogated cellular proliferation^48^. FOXM1 plays a pivotal role in promoting the expression of genes involved in DNA damage repair, while also contributing to the maintenance of cellular stemness and suppression of induced pluripotent stem cell reprogramming. FOXM1 is a key molecule that promotes epithelial-mesenchymal transition of tumor cells, and inhibition of its expression can effectively prevent cancer cell metastasis^49^. Analysis of gene expression in diverse clinical tumor samples has revealed up-regulation of FOXM1 expression in cancer cells, including melanoma. Heightened FOXM1 expression shows promise as a potential diagnostic and prognostic indicator for cancer. Consequently, FOXM1 suppression and regulation of downstream regulatory proteins are of utmost significance. Real-time PCR, Western blot, and immunofluorescence experiments validated the inhibitory impact of lasalocid on FOXM1 in melanoma cells. Lasalocid markedly restrained FOXM1 expression at both mRNA and protein levels in melanoma cells. To elucidate the mechanism underlying FOXM1 inhibition by lasalocid, we investigated its impact on FOXM1-regulated cell cycle progression, migration, invasion, and apoptosis-related proteins. After 24 h treatment with lasalocid in A375 and SK-MEL-28 cells, there was significant reduction in cyclinA2 and CDK2 as well as MMP2 and MMP9 expression levels. Simultaneously, lasalocid treatment reduced apoptosis inhibitory protein Bcl2 level and increased apoptosis-promoting protein BAX expression. The study results indicate lasalocid exerts its anti-melanoma effect by regulating expression of cell cycle, migratory invasion, and apoptosis-related proteins through the FOXM1 pathway.

The PI3K/AKT pathway is a pivotal cellular signaling cascade that governs diverse biological processes, including cell proliferation, survival, and metabolism. Activation of this pathway exerts stimulatory effects on melanoma cell proliferation and survival^43^. As a pivotal regulator within this pathway, AKT assumes a crucial role in cellular processes. Activation of AKT exerts multifaceted mechanisms to enhance melanoma cell survival by facilitating cell cycle progression and impeding apoptosis^50^. When the PI3K/AKT pathway is excessively activated, it hampers apoptosis and facilitates immune evasion of cancer cells, thereby fostering melanoma progression^51^. Aberrant activation of the PI3K/AKT pathway is also implicated in melanoma metastasis and invasion, as it promotes the epithelial-mesenchymal transition (EMT) of melanoma cells and enhances extracellular matrix degradation. Consequently, this activated pathway augments the metastatic potential and invasiveness of melanoma cells, thereby exacerbating its malignant phenotype^52^. Multiple studies have demonstrated that activation of the PI3K/AKT pathway confers resistance to conventional chemotherapeutic agents and targeted therapeutics in melanoma cells, thereby attenuating therapeutic efficacy^53^. Through analysis of sequencing data and review of FOXM1-related literature, we identified the PI3K/AKT pathway as a critical upstream regulatory pathway of FOXM1. Validating this, Western blot assays showed lasalocid significantly inhibits expression of phosphorylated PI3K and AKT (P-PI3K and P-AKT) with no effect on total PI3K and AKT proteins. Furthermore, stimulation with IGF1, a polypeptide activating PI3K/AKT, markedly upregulates P-PI3K and P-AKT without affecting total PI3K and AKT levels. Notably, IGF1-mediated PI3K/AKT activation also significantly increased FOXM1 protein expression. The CCK-8 assay confirmed IGF1-mediated PI3K/AKT activation promotes proliferation in A375 and SK-MEL-28 cells. Together, these findings suggest lasalocid down-regulates FOXM1 by inhibiting PI3K/AKT, impeding melanoma cell proliferation.

The JNK/P38 MAPK pathway represents a pivotal element of cellular signaling, exerting a crucial influence on the regulation of diverse biological processes such as cell growth, survival, apoptosis, inflammation, and other physiological responses^27^, ^54^. In melanoma, the JNK/P38 MAPK pathway plays a key role and is closely related to the occurrence, development and treatment of the disease^55^. Its activation status directly affects the proliferation and survival of melanoma cells. Activation of the JNK/P38 MAPK pathway induces apoptosis in melanoma cells and inhibits tumor growth^30^. Moreover, the activation of JNK/P38 MAPK pathway is intricately associated with melanoma cell metastasis and invasion. Studies have shown that activation of this pathway can hinder the invasive ability of melanoma cells and impede tumor dissemination^30^. A comprehensive analysis of sequencing data and FOXM1 literature revealed the JNK/P38 MAPK pathway as a critical regulatory pathway upstream of FOXM1. To validate JNK/P38 MAPK expression in human melanoma cells after 24 h of lasalocid treatment, Western blotting was performed. Results indicated lasalocid significantly induced P-P38 and P-JNK expression, but had no effect on total P38 and JNK levels. To block their respective signaling pathways, SB202190 and SP600125 were used. Inhibition of p38 MAPK by SB202190 led to significant down-regulation of P-P38 without affecting total P38. Similarly, inhibition of JNK MAPK by SP600125 resulted in significant decrease of P-JNK expression while total JNK remained unchanged. Notably, inhibition of both P38 and JNK MAPK pathways resulted in significant up-regulation of FOXM1 expression. Furthermore, CCK-8 assay detected substantial increase in proliferation for A375 and SK-MEL-28 cells following inhibition of P38/JNK MAPK pathways. Collectively, these findings suggest lasalocid suppresses melanoma cell proliferation by down-regulating FOXM1 through activating P38/JNK MAPK pathway.

## Conclusion

**Figure 9:**
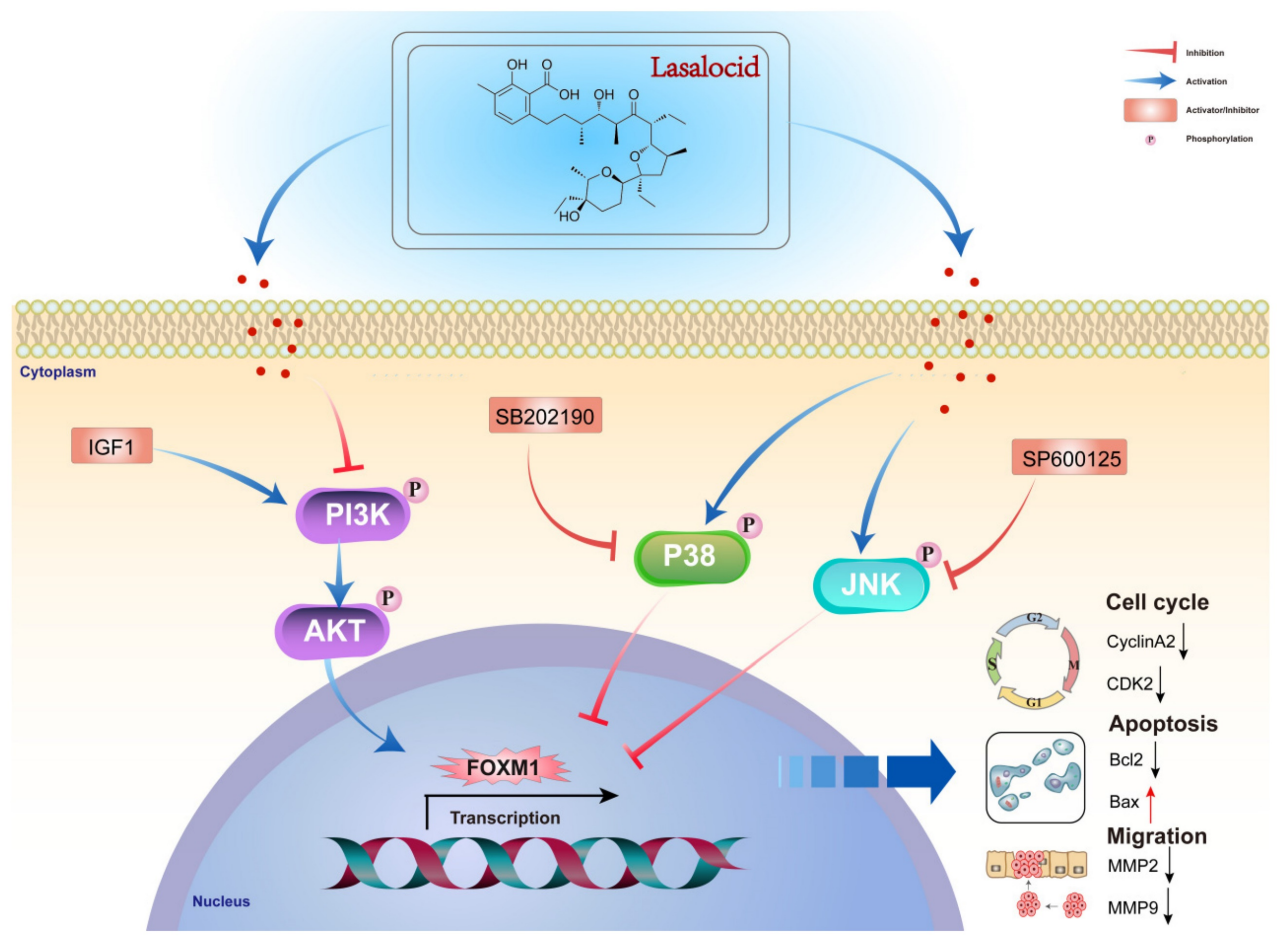
Diagram of the molecular mechanism by which lasalocid inhibits melanoma.

This study demonstrates that lasalocid inhibits the proliferation and migration of melanoma cells by downregulating FOXM1 through the PI3K/AKT and JNK/P38 MAPK pathways. These findings suggest that lasalocid may hold promise as a potential drug for the treatment of melanoma (Fig. 9).
